# Development of Dipicolylamine-Modified Cyclodextrins for the Design of Selective Guest-Responsive Receptors for ATP

**DOI:** 10.3390/molecules23030635

**Published:** 2018-03-12

**Authors:** Tatsuru Yamada, Shoji Fujiwara, Kyohhei Fujita, Yuji Tsuchido, Takeshi Hashimoto, Takashi Hayashita

**Affiliations:** 1 Department of Materials and Life Sciences, Faculty of Science and Technology, Sophia University, 7-1 Kioi-cho, Chiyoda, Tokyo 102-8554, Japan; tatsu614@eagle.sophia.ac.jp (T.Y.); s-fujiwara@sophia.ac.jp (S.F.); y-tsuchido@sophia.ac.jp (Y.T.); t-hasimo@sophia.ac.jp (T.H.); 2 Department of Current Legal Studies, Faculty of Law, Meiji Gakuin University, 1518 Kamikurata-cho, Totsuka-ku, Yokohama, Kanagawa 244-8539, Japan; 3 Graduate School of Medicine, The University of Tokyo, 7-3-1 Hongo, Bunkyo, Tokyo 113-0033, Japan; kfujita@m.u-tokyo.ac.jp

**Keywords:** supramolecular chemistry, cyclodextrin, ATP recognition, host–guest interaction, NMR analyses

## Abstract

The construction of supramolecular recognition systems based on specific host–guest interactions has been studied in order to design selective chemical sensors. In this study, guest-responsive receptors for ATP have been designed with cyclodextrins (CyDs) as a basic prototype of the turn-on type fluorescent indicator. We synthesized dipicolylamine (DPA)-modified CyD–Cu^2+^ complexes (**Cu·1*α***, **Cu·1*β***, and **Cu·1*γ***), and evaluated their recognition capabilities toward phosphoric acid derivatives in water. The UV-Vis absorption and fluorescence spectra revealed that **Cu·1*β*** selectively recognized ATP over other organic and inorganic phosphates, and that *β*-CyD had the most suitable cavity size for complexation with ATP. The 1D and 2D NMR analyses suggested that the ATP recognition was based on the host–guest interaction between the adenine moiety of ATP and the CyD cavity, as well as the recognition of phosphoric moieties by the Cu^2+^–DPA complex site. The specific interactions between the CyD cavity and the nucleobases enabled us to distinguish ATP from other nucleoside triphosphates, such as guanosine triphosphate (GTP), uridine triphosphate (UTP), and cytidine triphosphate (CTP). This study clarified the basic mechanisms of molecular recognition by modified CyDs, and suggested the potential for further application of CyDs in the design of highly selective supramolecular recognition systems for certain molecular targets in water.

## 1. Introduction

In the field of supramolecular chemistry, many kinds of molecular recognition systems have been developed that function by the specific interactions between host and guest molecules [1,2,3,4]. Cyclodextrins (CyDs), which are macrocyclic oligosaccharides composed of d-glucopyranose units, are well-known host molecules [5]. As CyDs include organic molecules in their hydrophobic cavities and increase the solubility and stability of the guest molecules in water, these molecules are utilized in various chemical fields, including analytical chemistry, organic chemistry, and biochemistry [6,7,8]. In addition, the application of specific host–guest interactions of chemically modified CyDs has resulted in such innovations as sensors, catalysts, functional materials, and carriers for drug delivery systems [9,10,11,12,13]. Therefore, it is very important to develop a design strategy to obtain selective CyD-based inclusion complexes. Recent reports of supramolecular recognition systems based on modified CyDs have revealed that CyDs possess the potential for use as highly selective receptors for various kinds of organic molecules [14,15,16]. For example, positively charged CyD bearing aminomethyl groups bound to adenosine monophosphate (AMP), adenosine diphosphate (ADP), or adenosine triphosphate (ATP) [17,18]. The affinity of these CyD hosts for nucleotides or nucleosides has been well investigated, although there are still few applications of CyDs to chemical sensors [19,20,21,22,23,24]. On the other hand, ATP recognition has been studied because of its significant roles in biological systems, and thus a number of ATP-targeting fluorescent probes have been reported over the last few decades [25,26,27]. However, there have been few examples of turn-on type chemical indicators that can distinguish nucleoside triphosphate from similar derivatives. Previously, we reported that Cu^2+^ complexes of dipicolylamine (DPA)-modified *γ*-CyD that possess an azobenzene unit selectively recognize ATP over other phosphoric acid derivatives [28]. The study demonstrated that multipoint recognition systems enable us to design selective receptors for certain organic molecules such as ATP. Here, we report a rational design strategy to obtain selective supramolecular recognition systems for ATP in water, where we used the affinity of chemically modified CyDs for nucleobases as a basic prototype of the turn-on type fluorescent indicators.

**Figure 1:**
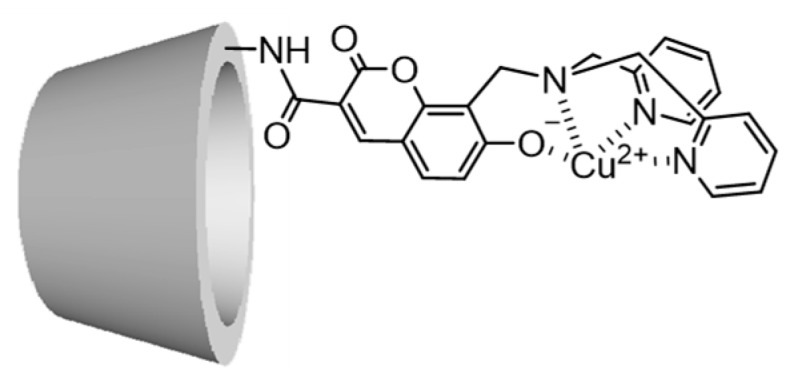
Structure of dipicolylamine-modified cyclodextrins (CyDs).

**Scheme 1:**
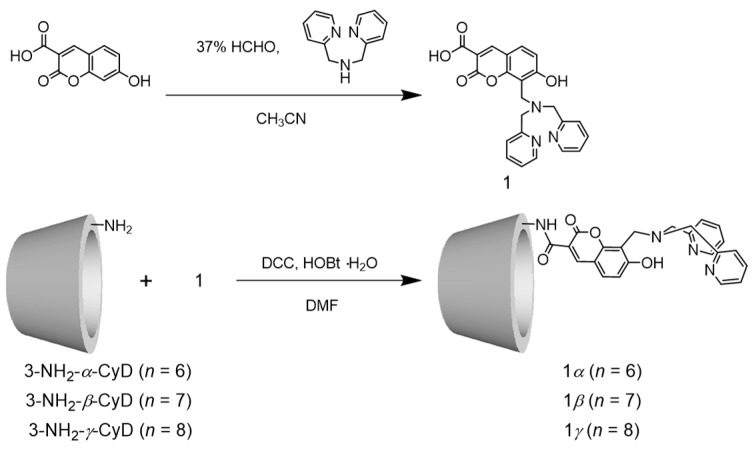
Synthesis of **1*α***, **1*β***, and **1*γ*** (*n*: the number of glucose units).

Our receptors (Figure 1) have two recognition sites: the DPA unit tethering with coumarin fluorophore (guest-targeting probe) and the CyD cavity. The DPA unit binds with heavy metal ions to form chelate complexes, and these metal complexes are known to recognize phosphoric acid derivatives [29]. The distance between the above two recognition sites was designed to enable selective host–guest interactions between the adenine moiety of ATP and the CyD cavity in addition to the recognition of phosphoric moieties by the Cu^2+^–DPA complex site. Although many studies use the Zn^2+^–DPA complex as the recognition site of phosphoric acid derivatives because of its high binding affinity, we dared to choose Cu^2+^ as the coordination center to design turn-on type fluorogenic receptors by the photo-induced electron transfer (PET) mechanism [30]. The Cu^2+^ complex of the guest-targeting probe is almost quenched but is activated by the coordination of the phosphoric moiety of ATP to the Cu^2+^–DPA complex site. We evaluated the recognition capabilities of the metal complexes of **1*α***, **1*β***, and **1*γ*** (Scheme 1) toward phosphoric acid derivatives. Three CyDs of different sizes were used to confirm the importance of the CyD cavity in this supramolecular recognition system.

## 2. Results and Discussion

### 2.1. Synthesis of ***1α***, ***1β***, and ***1γ***

Fluorescent probe **1**, which possesses coumarin as the fluorophore and DPA as the recognition site, was synthesized by the Mannich reaction [30]. Probe **1** and 3-NH_2_-(*α*, *β*, or ***γ***)-CyD were coupled by a condensation reaction [31]. The crude product was purified by reverse-phase column chromatography to obtain **1*α***, **1*β***, and **1*γ***. All the samples were identified by ^1^H NMR measurement and elemental analysis.

### 2.2. Metal Ion Recognition by ***1α***, ***1β***, and ***1γ***

**Figure 2:**
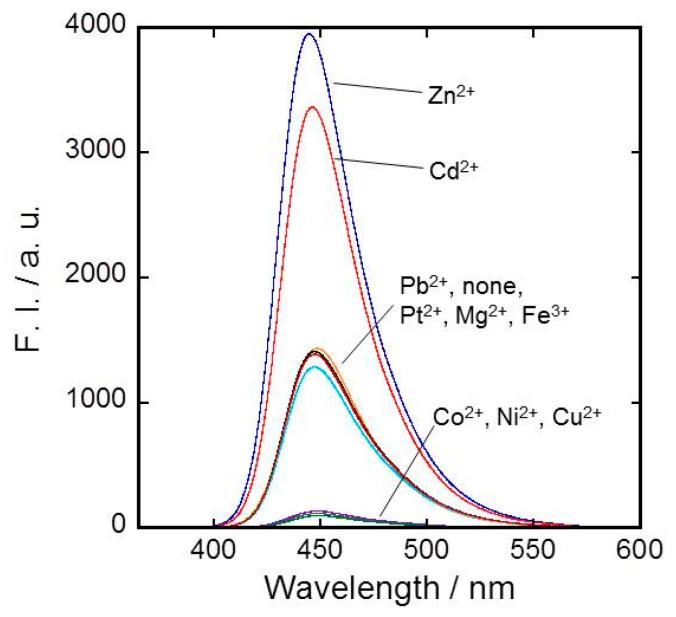
Fluorescence spectra of **1*β*** in the presence of metal ions in water, pH 6.4 adjusted by piperazine-1,4-bis(2-ethanesulfonic acid) (PIPES)/NaOH buffer, at 25 °C (*λ*_ex_ = 369 nm). [**1*β***] = 0.010 mM, [(Metal)(NO_3_)*_n_*] or [K_2_PtCl_4_] = 0.010 mM, [NaNO_3_] = 0.10 M (M = mol dm^−3^).

First, to evaluate the response function of modified CyDs toward metal ions, the fluorescence spectra of **1*α***, **1*β***, and **1*γ*** were measured in the presence of Mg^2+^, Fe^3+^, Co^2+^, Ni^2+^, Cu^2+^, Zn^2+^, Cd^2+^, Pt^2+^, and Pb^2+^. The addition of Zn^2+^ and Cd^2+^ possessing full d-orbitals enhanced the fluorescence intensity on the basis of the PET mechanism (Figure 2) [32]. On the other hand, the addition of Co^2+^, Ni^2+^, and Cu^2+^ possessing free d-orbitals quenched the fluorescence intensity due to the mechanisms of energy transfer (ET) or ligand to metal charge transfer (LMCT) [33,34]. Probes **1*α***, **1*β***, and **1*γ*** exhibited the same fluorescence behavior toward the metal ions, indicating that the CyD cavity has little influence on metal ion recognition. The complexation of the DPA unit to the metal ion was confirmed by metal ion titration experiments.

### 2.3. Phosphoric Acid Derivatives Recognitnion by **1α**, **1β**, and **1γ** Metal Ion Complexes

**Figure 3:**
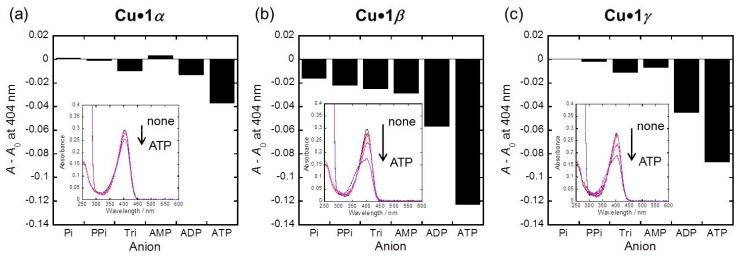
UV-Vis responses of (**a**) **Cu·1*α***; (**b**) **Cu·1*β***; and (**c**) **Cu·1*γ*** in the presence of phosphoric acid derivatives in water, pH 6.4 adjusted by PIPES/NaOH buffer, at 25 °C. [**1*α***, **1*β***, or **1*γ***] = 0.010 mM, [Cu(NO_3_)_2_] = 0.010 mM, [phosphoric acid derivative] = 1.0 mM.

**Figure 4:**
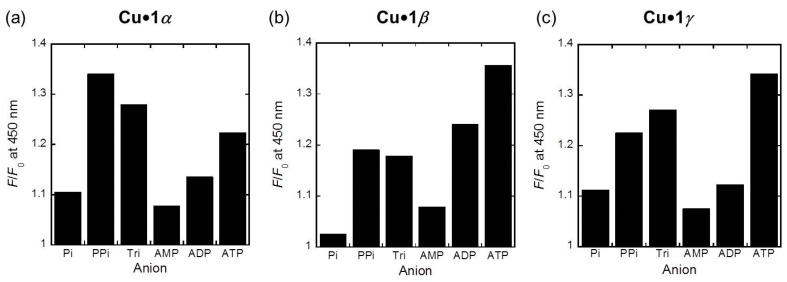
Fluorescence responses of (**a**) **Cu·1*α***, (**b**) **Cu·1*β***, and (**c**) **Cu·1*γ*** in the presence of phosphoric acid derivatives in water, pH 6.4 adjusted by PIPES/NaOH buffer, at 25 °C (*λ*_ex_ = 404, 408, 408 nm). [**1*α***, **1*β***, or **1*γ***] = 0.010 mM, [Cu(NO_3_)_2_] = 0.010 mM, [phosphoric acid derivative] = 1.0 mM.

In order to examine the recognition functions for phosphoric acid derivatives, the UV-Vis absorption spectra of **Cu·1*α***, **Cu·1*β***, and **Cu·1*γ*** were measured in the presence of monophosphate (Pi), pyrophosphate (PPi), triphosphate (Tri), adenosine monophosphate (AMP), adenosine diphosphate (ADP), and ATP. The addition of ATP markedly decreased the absorbance at 404 nm derived from the coumarin moiety (Figure 3). The coordination of phosphate anion to Cu^2+^ ion weakens the bond between the phenol oxygen atom and Cu^2+^ ion [35]. In addition, it is known that adenine interacts with aromatic compounds, including coumarin derivatives [36,37]. Both coordination and interaction are considered to change the characteristics of the electronic state on the coumarin moiety, which results in a specific absorbance change. The fluorescence spectra of **Cu·1*α***, **Cu·1*β***, and **Cu·1*γ*** were also examined to investigate the potential use of these molecules as fluorescent sensors. The fluorescence intensity at 450 nm was increased upon the addition of ATP (Figure 4). At first, the Cu^2+^ ion induced fluorescence quenching via ET or LMCT to the Cu^2+^ ion. However, the coordination of the phosphate anion to the Cu^2+^ ion weakened the bond between the DPA unit and the Cu^2+^ ion, which prevented the probe from inducing fluorescence quenching [38]. Therefore, the fluorescence intensity was recovered. In the presence of ATP, **Cu·1*β*** exhibited the most significant absorbance decrease and fluorescence enhancement. This selectivity for ATP suggested two mechanisms: the host–guest interaction between the adenine moiety of ATP and the CyD cavity, and the recognition of the phosphoric moiety of ATP by the Cu^2+^–DPA complex site. The host–guest interaction stabilized supramolecular complex ATP/**Cu·1*β***, which led to the specific response. These results indicated that the CyD cavity played an important role in the recognition system.

We also evaluated the UV-Vis absorption spectra and the fluorescence spectra of Zn^2+^ complexes **Zn·1*α***, **Zn·1*β***, and **Zn·1*γ***. The absorbance of **Zn·1*α***, **Zn·1*β***, and **Zn·1*γ*** was decreased upon the addition of ATP but these responses were weaker than those of the Cu^2+^ complexes. The addition of ATP decreased the fluorescence intensity of **Zn·1*α***, **Zn·1*β***, and **Zn·1*γ***. The Zn^2+^ complexes exhibit fluorescence emission by suppressing the intramolecular PET mechanism. The coordination of the phosphate anion to the Zn^2+^ ion weakens the bond between the DPA unit and the Zn^2+^ ion, thereby inducing fluorescence quenching. However, the quenching was not suitable for our strategy because we wanted to focus on the application to turn-on type indicators.

To compare the complex formation constants to ATP, the UV-Vis absorption spectra of **Cu·1*α***, **Cu·1*β***, and **Cu·1*γ*** in the presence of various concentrations of ATP were measured. The complex formation constants of **Cu·1*α***, **Cu·1*β***, and **Cu·1*γ*** to ATP were determined to be (1.7 ± 0.06) × 10^3^ M^−1^, (3.3 ± 0.15) × 10^3^ M^−1^, and (2.3 ± 0.12) × 10^3^ M^−1^, respectively, by the Benesi–Hildebrand method [39]. These complex formation constants confirmed that **Cu·1*β*** formed the most stable supramolecular complex with ATP in water.

**Figure 5:**
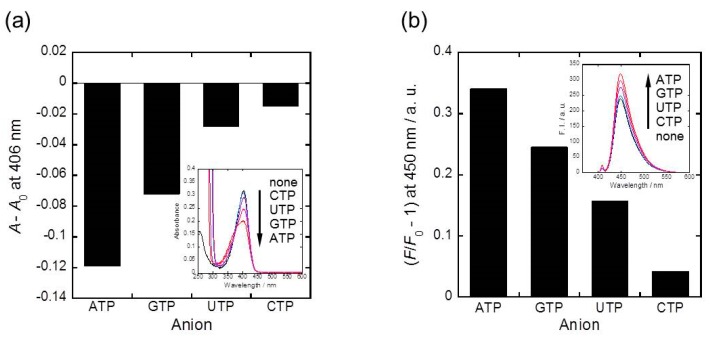
(**a**) UV-Vis and (**b**) Fluorescence responses of **Cu·1*β*** in the presence of nucleoside triphosphates in water, pH 6.4 adjusted by PIPES/NaOH buffer, at 25 °C (*λ*_ex_ = 408 nm). [**1*β***] = 0.010 mM, [Cu(NO_3_)_2_] = 0.010 mM, [nucleoside triphosphate] = 1.0 mM.

The UV-Vis and fluorescence responses revealed that **Cu·1*β*** selectively recognized ATP over other phosphoric acid derivatives. In order to evaluate the recognition selectivity of **Cu·1*β*** for nucleoside triphosphates, UV-Vis absorption spectra and fluorescence spectra were measured in the presence of each nucleoside triphosphate: ATP, guanosine triphosphate (GTP), uridine triphosphate (UTP), and cytidine triphosphate (CTP). The addition of each nucleoside triphosphate decreased the absorbance and enhanced the fluorescence intensity of **Cu·1*β*** (Figure 5). In particular, **Cu·1*β*** selectively recognized ATP over the other nucleoside triphosphates. This selectivity is considered to be based on the host–guest interaction between the nucleobase and the CyD cavity. Adenine is known to interact well with the CyD cavity compared with the other nucleobases [17]. This result also disclosed that the CyD cavity is involved in the recognition. Moreover, **Cu·1*β*** selectively recognized a purine base over a pyrimidine base, indicating the potential for use as receptors capable of distinguishing between purine and pyrimidine type nucleoside triphosphates.

### 2.4. 1D and 2D NMR Analyses

**Figure 6:**
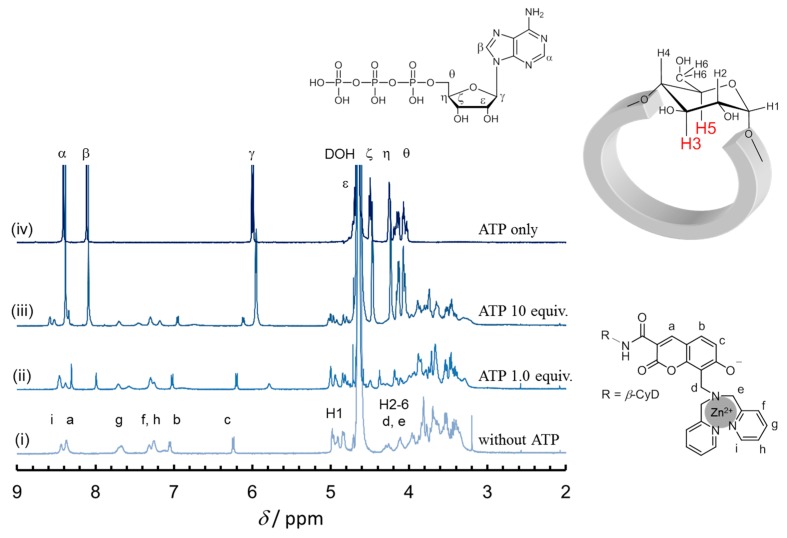
^1^H NMR spectra of complex ATP/**Zn·1*β*** and ATP (500 MHz, solvent: D_2_O). (**i**) [**Zn·1*β***] = 0.5 mM, (**ii**) [**Zn·1*β***] = 0.5 mM, [ATP] = 0.5 mM, (**iii**) [**Zn·1*β***] = 0.5 mM, [ATP] = 5.0 mM, (**iv**) [ATP] = 10 mM.

In order to clarify the coordination and interaction between **Cu·1*β*** and ATP, the ^1^H NMR spectra of complex **Zn·1*β*** were measured in the presence and absence of ATP. In general, it is difficult to evaluate the NMR spectra of the Cu^2+^ complex owing to the paramagnetism of the Cu^2+^ ion. Upon the addition of ATP, shifts of proton peaks assigned to **h** and **i** derived from the DPA site were noted (Figure 6), indicating that the Zn^2+^–DPA complex site recognized the phosphoric moiety of ATP [40,41,42]. Shifts of proton peaks assigned to **b** and **c** derived from the coumarin moiety were also observed, revealing that the hydroxyl group is associated with the coordination to Zn^2+^ ion.

**Figure 7:**
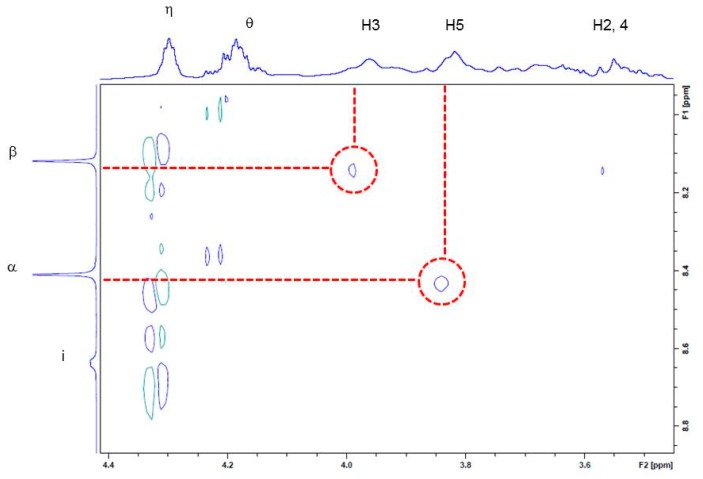
NOESY spectrum of complex ATP/**Zn·1*β*** (400 MHz, solvent: D_2_O). [**Zn·1*β***] = 5.0 mM, [ATP] = 25 mM.

We also analyzed the supramolecular conformation of the ATP/**Cu·1*β*** complex by conducting nuclear Overhauser effect spectroscopy (NOESY) measurements of complex **Cu·1*β.*** In the NOESY measurements, protons in close proximity to each other give rise to correlation peaks [43,44]. Correlations between the protons derived from the adenine moiety of ATP (α and β) and the protons derived from the inside cavity of CyD (H3 and H5) were observed (Figure 7). The results suggested that the adenine moiety interacted with the inside cavity of CyD due to host–guest interactions. If the NOESY spectra of **Cu·1*β*** could be measured, much stronger correlations would be expected because the Cu^2+^ complex exhibited the higher binding ability to ATP.

### 2.5. Suggested Supramolecular Conformation

**Figure 8:**
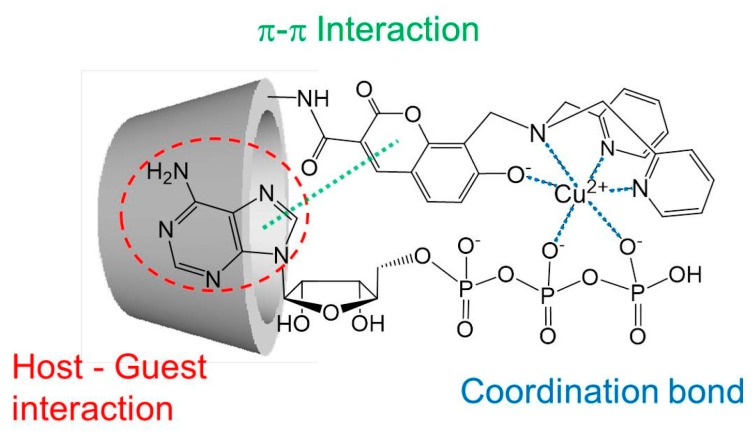
Suggested supramolecular conformation of the ATP/**Cu·1*β*** complex.

To sum up the obtained results, the supramolecular conformation shown in Figure 8 is suggested. First, the coordination of the DPA site and the hydroxyl group to the metal ion was confirmed by UV-Vis and ^1^H NMR measurements. Secondly, the coordination of the phosphoric moiety of ATP to the metal ion–DPA complex site was revealed by ^1^H NMR experiments. Thirdly, the interaction between the adenine moiety of ATP and the coumarin fluorophore was implied by the UV-Vis responses, although this interaction was weak. Finally, the host–guest interaction between the adenine moiety and the CyD cavity was suggested by NOESY measurements. Thus, **Cu·1*β*** was found to recognize ATP through the host–guest interaction between the adenine moiety and the CyD cavity in addition to the recognition of the phosphoric moiety by the metal ion–DPA complex site. In particular, the cavity size of CyD is a major contributor to the difference in selectivity for ATP. Therefore, the selective ATP recognition in this system is considered to markedly depend on the multipoint interaction in water.

## 3. Materials and Methods

### 3.1. Reagents

All organic solvents and reagents including 3-NH_2_-CyDs were commercially available with guaranteed grades and used without further purification. Water was doubly distilled and deionized by a Milli-Q water system before use.

### 3.2. Apparatus

UV-Vis absorption spectra were measured with a UV-Vis spectrophotometer equipped with a Peltier thermocontroller and a 10 mm quartz cell. Fluorescence spectra were measured with a fluorescence spectrometer with a 10 mm quartz cell.

### 3.3. Metal Ion Recognition by ***1α***, ***1β***, and ***1γ***

To evaluate the metal ion recognition ability of **1*α***, **1*β***, and **1*γ***, fluorescence spectral measurements were performed. Solutions containing **1*α***, **1*β***, and **1*γ*** (0.010 mM), metal ion (0.010 mM), and piperazine-1,4-bis(2-ethanesulfonic acid) (PIPES)/NaOH buffer (5.0 mM, pH 6.4, adjusted using NaOH) were prepared and spectra were recorded at 25 °C.

### 3.4. Phosphoric Acid Derivatives Recognitnion by ***1α***, ***1β***, and ***1γ*** Metal Ion Complexes

To evaluate the phosphoric acid recognition ability of **1*α***, **1*β***, and **1*γ*** metal ion complexes, UV-Vis absorption and fluorescence spectral measurements were performed. Solutions containing **1*α***, **1*β***, and **1*γ*** (0.010 mM), Cu(NO_3_)_2_ or Zn(NO_3_)_2_ (0.010 mM), phosphoric acid derivatives (1.0 mM, pH 7.4, adjusted using NaOH), and PIPES/NaOH buffer (10 mM, pH 6.4, adjusted using NaOH) were prepared and spectra were recorded at 25 °C.

### 3.5. Calculation of Binding Constants of ***Cu·1α***, ***Cu·1β***, and ***Cu·1γ*** to ATP

To calculate the binding constants of **Cu·1*α***, **Cu·1*β***, and **Cu·1*γ*** to ATP, UV-Vis absorption spectral measurements were performed. Solutions containing **1*α***, **1*β***, and **1*γ*** (0.010 mM), Cu(NO_3_)_2_ (0.010 mM), and PIPES/NaOH buffer (10 mM, pH 6.4, adjusted using NaOH) were prepared and spectra were recorded at 25 °C, while ATP concentrations varied from 0 to 4.0 mM. The binding constants were calculated by the Benesi–Hildebrand method.

### 3.6. NOESY Measurements of ***Zn·1β***/ATP Complex

To evaluate the supramolecular conformation of the **Cu·1*β***/ATP complex, NOESY spectral measurements of the **Zn·1*β***/ATP complex were performed. Solutions containing **1*β*** (5.0 mM), Zn(NO_3_)_2_ (5.0 mM), and ATP (25 mM, pD 7, adjusted using NaOD) were prepared and NOESY spectra were recorded.

### 3.7. General Procedure for the Synthesis of ***1***

Aqueous formaldehyde (305 mg, 3.76 mmol) was added to a solution of DPA (729 mg, 3.66 mmol) in 15 cm^3^ of acetonitrile. After 3 h of refluxing, 7-hydroxycoumarin-3-carboxylic acid (499 mg, 2.42 mmol) dissolved in 30 cm^3^ of acetonitrile was added dropwise. The entire reaction mixture was stirred at 40 °C for 21 h. The yellow precipitate was filtered and washed with acetonitrile. The yellow solid was washed with water and extracted with chloroform, and the extract was concentrated to obtain **1** as a yellow solid in 71% yield. The sample was identified by ^1^H NMR and FAB-MS. The characterization data are shown in the Supplementary Materials (Figures S1 and S2, and Table S1).

### 3.8. General Procedure for the Synthesis of ***1α***, ***1β***, and ***1γ***

A mixture of **1**, *N*,*N′*-dicyclohexylcarbodiimide (DCC), and 1-hydroxybenzotriazole (HOBt)**·**H_2_O (Table 1) in 4.0 cm^3^ of super dehydrated *N*,*N*-dimethylformamide (DMF) was stirred in an ice bath for 20 min. The compound 3-NH_2_-(*α*, *β*, or *γ*)-CyD in 2.0 cm^3^ of super dehydrated DMF was added dropwise over a period of 5 min. The entire reaction mixture was again stirred in an ice bath for 30 min and then at room temperature for 24 h. The solution was distilled under reduced pressure until half of the DMF was removed. The sample was kept in a refrigerator for one day. The resulting white solid was removed by filtration. The filtrate was poured into 800 cm^3^ of acetone and stirred for 1 h to obtain a yellow precipitate. The crude product was charged on a column of CHROMATOREX ODS-DM1020T (reverse phase) and eluted with 10% acetonitrile-90% water (*v/v*, 1.0 dm^3^) and then with 20% acetonitrile-80% water (*v/v*, 0.5 dm^3^). The second spot was collected as the desired product. The fraction was lyophilized under reduced pressure to obtain a yellow solid. The compounds **1*α***, **1*β***, and **1*γ*** were obtained in 52%, 46%, and 49% yield, respectively. All the samples were identified by ^1^H NMR measurement and elemental analysis. The characterization data are shown in the Supplementary Materials (Figures S3–S5 and Tables S2–S7).

## 4. Conclusions

We have developed fluorogenic DPA-modified CyDs to obtain supramolecular recognition systems for ATP as a basic prototype of the selective guest-responsive indicator. The absorption and fluorescence responses revealed that **Cu·1*β*** recognized ATP selectively over other phosphoric acid derivatives possessing similar phosphoric moieties or nucleobases. The *β*-CyD cavity was found to be the most acceptable for the formation of the supramolecular complex with ATP. NMR analysis suggested that the selective ATP recognition not only depended on the recognition of phosphoric moieties by the Cu^2+^–DPA complex site but also on the host–guest interactions between the adenine moiety of ATP and the CyD cavity. The results demonstrated that the CyD cavity played an important role in the selectivity over other nucleoside triphosphates in the supramolecular recognition system. Therefore, this system enabled us to discriminate a purine base from a pyrimidine base as well as the lengths of the phosphoric moieties. This study clarified the basic mechanisms of modified CyDs as selective indicators, and suggested the potential for further application in the design of supramolecular recognition systems based on CyDs with high selectivity for certain molecular targets in water.

## Figures and Tables

**Table 1:** The reagents used to synthesize **1*α***, **1*β***, and **1*γ***.

Compound	1	DCC	HOBt·H_2_O	3-NH_2_-(*𝛼*, *𝛽*, or *γ*)-CyD
**1*α***	44 mg, 0.11 mmol	24 mg, 0.11 mmol	18 mg, 0.12 mmol	70 mg, 0.072 mmol
**1*β***	56 mg, 0.13 mmol	28 mg, 0.14 mmol	21 mg, 0.14 mmol	102 mg, 0.090 mmol
**1*γ***	42 mg, 0.10 mmol	22 mg, 0.11 mmol	16 mg, 0.10 mmol	82 mg, 0.063 mmol

## Supplementary Materials

Supplementary materials are available online. Compound characterization data, the pH profiles, phosphoric acid recognition by UV-Vis and fluorescence measurements of **1*α***, **1*β***, or **1*γ*** metal ion complexes, calculation of binding constants, competitive experiments, and copies of ^1^H-^1^H COSY and NOESY spectra of **Zn·1*β***/ATP complex.
