# Supplementary material for: Development of Dipicolylamine-Modified Cyclodextrins for the Design of Selective Guest-Responsive Receptors for ATP
Source: Molecules. 2018 Mar 12;23(3):635. doi: 10.3390/molecules23030635 (PMC6017074; doi:10.3390/molecules23030635)
Supplement: Supplementary file 1 [file molecules-23-00635-s001.docx]

Supplementary Materials

Development of Dipicolylamine-Modified Cyclodextrins for the Design of Selective Guest-Responsive Receptors for ATP

Tatsuru Yamada ^1^, Shoji Fujiwara ^1^, Kyohhei Fujita ^2^, Yuji Tsuchido ^1^, Takeshi Hashimoto ^1^ and Takashi Hayashita ^1,^*

^1^ Department of Materials and Life Sciences, Faculty of Science and Technology, Sophia University, 7-1 Kioi-cho, Chiyoda, Tokyo 102-8554, Japan. Fax: +81-3-3238-3361; Tel: +81-3-3238-3372; E-mail: ta-hayas@sophia.ac.jp

^2^ Graduate School of Medicine, The University of Tokyo, 7-3-1, Hongo, Bunkyo, Tokyo 113-0033, Japan

**Table of Contents:**

1. **Characterization data 3**
2. **The pH profiles of 1*α*, 1*β*, and 1*γ*  9**
3. **UV-vis absorption spectra of 1*α*, 1*β*, and 1*γ* in the presence of metal ions 13**
4. **Fluorescence spectra of 1*α* and 1*γ* in the presence of metal ions 14**
5. **Binding constants of 1*α*, 1*β*, and 1*γ* to Cu^2+^ 15**
6. **Fluorescence spectra of Cu•1*α*, Cu•1*β*, and Cu•1*γ* in the presence of phosphate anions**

**17**

1. **UV-vis absorption spectra of Zn•1*α*, Zn•1*β*, and Zn•1*γ* in the presence of phosphate anions 19**
2. **Fluorescence spectra of Zn•1*α*, Zn•1*β*, and Zn•1*γ* in the presence of phosphate anions**

**21**

1. **Complex formation constants of Cu•1*α*, Cu•1*β*, and Cu•1*γ* to ATP 23**
2. **The competitive binding experiments of Cu•1*β* 25**
3. **NMR analyses of Zn•1*β* 26**

**1. Characterization data.**


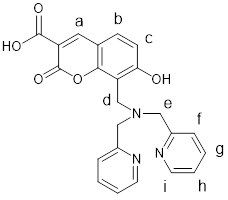


CHCl_3_

e

d

d

a

i

c

h

f

b

g

**Figure S1.** ^1^H NMR spectrum of **1** (300 MHz, solvent: CDCl_3_).

**Table S1.** ^1^H NMR assignment of **1**.

| *δ* / ppm | Assignment | Intensity | | Splitting |
| --- | --- | --- | --- | --- |
|  |  | Calcd. | Found |  |
| 3.96 | e | 4H | 3.92H | s |
| 4.14 | d | 2H | 2.04H | s |
| 7.02 | c | 1H | 1.09H | d |
| 7.21 | h | 2H | 2.11H | t |
| 7.33 | f | 2H | 2.07H | d |
| 7.53 | b | 1H | 1.09H | d |
| 7.67 | g | 2H | 2.08H | td |
| 8.57 | i | 2H | 1.99H | d |
| 8.79 | a | 1H | 1.00H | s |

Calcd. for C_23_H_20_N_3_O_5_: [M + H]^+^ = 418.13, Found: 418.1.

Mass-to-charge ratio (*m* / *z*)

**Figure S2.** FAB-MS spectrum of **1**.

H1

d, e, H2-H6

DOH

a

i

g

h

c

b,f

**Figure S3.** ^1^H NMR spectrum of **1*α*** (300 MHz, solvent: D_2_O).

**Table S2.** ^1^H NMR assignment of **1*α***.

| *δ* / ppm | Assignment | Intensity | | Splitting | Coupling Constant / Hz |
| --- | --- | --- | --- | --- | --- |
|  |  | Calcd. | Found |  |  |
| 3.96 | e | 4H | 3.92H | s | ‐ |
| 4.14 | d | 2H | 2.04H | s | ‐ |
| 7.02 | c | 1H | 1.09H | d | *J*_cb_ = 8.72 |
| 7.21 | h | 2H | 2.11H | t | *J*_hi,_ *J*_hg_ = 3.76, 6.46 |
| 7.33 | f | 2H | 2.07H | d | *J*_fg_ = 7.81 |
| 7.53 | b | 1H | 1.09H | d | *J*_bc_ = 8.72 |
| 7.67 | g | 2H | 2.08H | td | *J*_gf,_ *J*_gh_ = 7.81, 7.66 |
| 8.57 | i | 2H | 1.99H | d | *J*_ih_ = 3.91 |
| 8.79 | a | 1H | 1.00H | s | *-* |

H1

d, H2-H6

DOH

a, i

g

h

c

b,f

e

**Figure S4.** ^1^H NMR spectrum of **1*β*** (300 MHz, solvent: D_2_O).

**Table S3.** ^1^H NMR assignment of **1*β***.

| *δ* / ppm | Assignment | Intensity | | Splitting |
| --- | --- | --- | --- | --- |
|  |  | Calcd. | Found |  |
| 3.27-4.04 | H2-H6, d | 44H | 44.6H | m |
| 4.31 | e | 4H | 3.45H | s |
| 4.79-4.97 | H1 | 7H | 6.98H | m |
| 6.49 | c | 1H | 0.99H | d |
| 7.11 | h | 2H | 1.97H | t |
| 7.26 | b, f | 3H | 3.08H | d |
| 7.53 | g | 2H | 2.04H | t |
| 8.29 | a, i | 3H | 3.00H | m |

H1

d, e, H2-H6

DOH

a

i

g,b,f

h

c

**Figure S5.** ^1^H NMR spectrum of **1*γ*** (300 MHz, solvent: D_2_O).

**Table S4.** ^1^H NMR assignment of **1*γ***.

| *δ* / ppm | Assignment | Intensity | | Splitting |
| --- | --- | --- | --- | --- |
|  |  | Calcd. | Found |  |
| 2.70-4.18 | H2-H6, e, d | 54H | 52.6H | m |
| 4.74-4.99 | H1 | 8H | 8.00H | m |
| 6.67 | c | 1H | 1.41H | d |
| 7.00 | h | 2H | 2.30H | t |
| 7.24-7.36 | f, b, g | 5H | 5.12H | d, m |
| 8.07 | i | 2H | 2.33H | d |
| 8.38 | a | 1H | 1.28H | s |

**Table S5.** Elemental analysis of **1*α*** (C_59_H_78_N_4_O_33_).

|  | C / % | H / % | N / % | C / N |
| --- | --- | --- | --- | --- |
| Calcd. | 51.68 | 5.73 | 4.09 | 12.6 |
| Found | 45.23 | 5.88 | 3.61 | 12.5 |
| + 10H_2_O | 45.68 | 6.37 | 3.61 | 12.7 |

**Table S6.** Elemental analysis of **1*β*** (C_65_H_88_N_4_O_38_).

|  | C / % | H / % | N / % | C / N |
| --- | --- | --- | --- | --- |
| Calcd. | 50.91 | 5.78 | 3.65 | 13.9 |
| Found | 44.40 | 6.34 | 3.11 | 14.3 |
| + 12H_2_O | 44.62 | 6.45 | 3.20 | 13.9 |

**Table S7.** Elemental analysis of **1*γ*** (C_71_H_98_N_4_O_43_).

|  | C / % | H / % | N / % | C / N |
| --- | --- | --- | --- | --- |
| Calcd. | 50.30 | 5.83 | 3.30 | 15.2 |
| Found | 46.38 | 6.07 | 2.98 | 15.6 |
| +8H_2_O | 46.36 | 6.25 | 3.05 | 15.2 |

**2. The pH profiles of 1*α*, 1*β*, and 1*γ***

(a)

pH 11

pH 2

(b)

pH 11

pH 2

(c)

pH 2

pH 11

**Figure S6.** UV-vis absorption spectra of (a) **1*α***, (b) **1*β***, and (c) **1*γ*** with pH changes in water, adjusted by HEPES/NaOH buffer, at 25ºC. [**1*α***, **1*β***, or **1*γ***] = 1.0 × 10^-5^ M, [NaNO_3_] = 0.10 M.

pH 6.7

(a)

pH 2.0

(b)

pH 12

pH 7.0

(c)

**Figure S7.** (a), (b) Fluorescence spectra and (c) fluorescence intensity at 449 nm for **1*α*** (*λ*_ex_ = 375 nm) with pH changes in water, adjusted by HEPES/NaOH buffer, at 25ºC. [**1*α***] = 1.0 × 10^-5^ M, [NaNO_3_] = 0.10 M.

(a)

pH 6.7

pH 2.0

(b)

pH 12

pH 7.0

(c)

**Figure S8.** (a), (b) Fluorescence spectra and (c) fluorescence intensity at 447 nm for **1*β*** (*λ*_ex_ = 375 nm) with pH changes in water, adjusted by HEPES/NaOH buffer, at 25ºC. [**1*β***] = 1.0 × 10^-5^ M, [NaNO_3_] = 0.10 M.

pH 2.0

pH 6.0

(a)

(b)

pH 12

pH 6.3

(c)

**Figure S9.** (a), (b) Fluorescence spectra and (c) fluorescence intensity at 450 nm for **1*γ*** (*λ*_ex_ = 367 nm) with pH changes in water, adjusted by HEPES/NaOH buffer, at 25ºC. [**1*γ***] = 1.0 × 10^-5^ M, [NaNO_3_] = 0.10 M.

3. **UV-vis absorption spectra of 1*α*, 1*β*, and 1*γ* in the presence of metal ions**

Co^2+^, Ni^2+^, Cu^2+^, Zn^2+^, Cd^2+^, Pb^2+^

none, Mg^2+^, Fe^3+^, Pt^2+^

(a)

(b)

none, Mg^2+^, Fe^3+^, Pt^2+^

Co^2+^, Ni^2+^, Cu^2+^, Zn^2+^, Cd^2+^, Pb^2+^

(c)

none, Mg^2+^, Fe^3+^, Pt^2+^

Co^2+^, Ni^2+^, Cu^2+^, Zn^2+^, Cd^2+^, Pb^2+^

**Figure S10.** UV-Vis absorption spectra of (a) **1*α***, (b) **1*β***, and (c) **1*γ*** in the presence of metal ions in water, pH 6.4 adjusted by PIPES/NaOH buffer, at 25ºC. [**1*α***, **1*β***, or **1*γ***], [(metal)(NO_3_)_n_], [K_2_PtCl_4_] = 1.0 × 10^-5^ M, [NaNO_3_] = 0.10 M.

**4. Fluorescence spectra of 1*α* and 1*γ* in the presence of metal ions**

(a)

Zn^2+^

Cd^2+^

none

Mg^2+^

Pt^2+^

Fe^3+^

Pb^2+^

Ni^2+^

Co^2+^

Cu^2+^

(b)

Zn^2+^

Cd^2+^

Pb^2+^

Pt^2+^

Fe^3+^

Mg^2+^

none

Ni^2+^

Co^2+^

Cu^2+^

**Figure S11.** Fluorescence spectra of (a) **1*α*** and (b) **1*γ*** in the presence of metal ions in water, pH 6.4 adjusted by PIPES/NaOH buffer, at 25ºC (*λ*_ex_ = 374 nm). [**1*α*** or **1*γ***], [(metal)(NO_3_)_2_], [K_2_PtCl_4_] = 1.0 × 10^-5^ M, [NaNO_3_] = 0.10 M.

**5. Binding constants of 1*α*, 1*β*, and 1*γ* to Cu^2+^**

0 equiv.

4 equiv.

(b)

(a)

**Figure S12.** (a) UV-Vis absorption spectra and (b) titration curve of **1*α*** in water, pH 6.4 adjusted by PIPES/NaOH buffer, at 25ºC. [**1*α***] = 1.0 × 10^-5^ M, [Cu(NO_3_)_2_] = 0 - 4.0 × 10^-5^ M, [NaNO_3_] = 0.10 M.

(b)

(a)

0 equiv.

4 equiv.

**Figure S13.** (a) UV-Vis absorption spectra and (b) titration curve of **1*β*** in water, pH 6.4 adjusted by PIPES/NaOH buffer, at 25ºC. [**1*β***] = 1.0 × 10^-5^ M, [Cu(NO_3_)_2_] = 0 - 4.0 × 10^-5^ M, [NaNO_3_] = 0.10 M.

(a)

(b)

4 equiv.

0 equiv.

**Figure S14.** (a) UV-Vis absorption spectra and (b) titration curve of **1*γ*** in water, pH 6.4 adjusted by PIPES/NaOH buffer, at 25ºC. [**1*γ***] = 1.0 × 10^-5^ M, [Cu(NO_3_)_2_] = 0 - 4.0 × 10^-5^ M, [NaNO_3_] = 0.10 M.

**Table S8.** Binding constants of **1*α***, **1*β***, and **1*γ*** to Cu^2+^.

| Probe | Binding Constants / M^-1^ |
| --- | --- |
| **1*α*** | > 10^8^ (ND) |
| **1*β*** | > 10^8^ (ND) |
| **1*γ*** | > 10^8^ (ND) |

**6. Fluorescence spectra of Cu•1*α*, Cu•1*β*, and Cu•1*γ* in the presence of phosphate anions.**

**Figure S15.** Fluorescence spectra of **Cu•1*α*** in the presence of phosphate anions in water, pH 6.4 adjusted by PIPES/NaOH buffer, at 25ºC (*λ*_ex_ = 404 nm). [**1*α***] = 0.010 mM, [Cu(NO_3_)_2_] = 0.010 mM, [phosphate anion] = 1.0 mM.

**Figure S16.** Fluorescence spectra of **Cu•1*β*** in the presence of phosphate anions in water, pH 6.4 adjusted by PIPES/NaOH buffer, at 25ºC (*λ*_ex_ = 408 nm). [**1*β***] = 0.010 mM, [Cu(NO_3_)_2_] = 0.010 mM, [phosphate anion] = 1.0 mM.

**Figure S17.** Fluorescence spectra of **Cu•1*γ*** in the presence of phosphate anions in water, pH 6.4 adjusted by PIPES/NaOH buffer, at 25ºC (*λ*_ex_ = 408 nm). [**1*γ***] = 0.010 mM, [Cu(NO_3_)_2_] = 0.010 mM, [phosphate anion] = 1.0 mM.

**7. UV-vis absorption spectra of Zn•1*α*, Zn•1*β*, and Zn•1*γ* in the presence of phosphate anions.**

**Figure S18.** UV-vis absorption responses of **Zn•1*α*** in the presence of phosphate anions in water, pH 6.4 adjusted by PIPES/NaOH buffer, at 25ºC. [**1*α***] = 0.010 mM, [Zn(NO_3_)_2_] = 0.010 mM, [phosphate anion] = 1.0 mM.

**Figure S19.** UV-vis absorption responses of **Zn•1*β*** in the presence of phosphate anions in water, pH 6.4 adjusted by PIPES/NaOH buffer, at 25ºC. [**1*β***] = 0.010 mM, [Zn(NO_3_)_2_] = 0.010 mM, [phosphate anion] = 1.0 mM.

**Figure S20.** UV-vis absorption responses of **Zn•1*γ*** in the presence of phosphate anions in water, pH 6.4 adjusted by PIPES/NaOH buffer, at 25ºC. [**1*γ***] = 0.010 mM, [Zn(NO_3_)_2_] = 0.010 mM, [phosphate anion] = 1.0 mM.

**8. Fluorescence spectra of Zn•1*α*, Zn•1*β*, and Zn•1*γ* in the presence of phosphate anions.**

**Figure S21.** Fluorescence responses of **Zn•1*α*** in the presence of phosphate anions in water, pH 6.4 adjusted by PIPES/NaOH buffer, at 25ºC (*λ*_ex_ = 375 nm). [**1*α***] = 0.010 mM, [Zn(NO_3_)_2_] = 0.010 mM, [phosphate anion] = 1.0 mM.

**Figure S22.** Fluorescence responses of **Zn•1*β*** in the presence of phosphate anions in water, pH 6.4 adjusted by PIPES/NaOH buffer, at 25ºC (*λ*_ex_ = 408 nm). [**1*β***] = 0.010 mM, [Zn(NO_3_)_2_] = 0.010 mM, [phosphate anion] = 1.0 mM.

**Figure S23.** Fluorescence responses of **Zn•1*γ*** in the presence of phosphate anions in water, pH 6.4 adjusted by PIPES/NaOH buffer, at 25ºC (*λ*_ex_ = 380 nm). [**1*γ***] = 0.010 mM, [Zn(NO_3_)_2_] = 0.010 mM, [phosphate anion] = 1.0 mM.

**9. Complex formation constants of Cu•1*α*, Cu•1*β*, and Cu•1*γ* to ATP.**

(a)

(b)

0 equiv.

400 equiv.

**Figure S24.** (a) UV-Vis absorption spectra and (b) Benesi-Hildebrand plot of **Cu•1*α*** in water, pH 6.4 adjusted by PIPES/NaOH buffer, at 25ºC. [**1*α***] = 0.010 mM, [Cu(NO_3_)_2_] = 0.010 mM, [ATP] = 0 - 4.0 mM.

(a)

(b)

0 equiv.

400 equiv.

**Figure S25.** (a) UV-Vis absorption spectra and (b) Benesi-Hildebrand plot of **Cu•1*β*** in water, pH 6.4 adjusted by PIPES/NaOH buffer, at 25ºC. [**1*β***] = 0.010 mM, [Cu(NO_3_)_2_] = 0.010 mM, [ATP] = 0 - 4.0 mM.

(b)

(a)

0 equiv.

400 equiv.

**Figure S26.** (a) UV-Vis absorption spectra and (b) Benesi-Hildebrand plot of **Cu•1*γ*** in water, pH 6.4 adjusted by PIPES/NaOH buffer, at 25ºC. [**1*γ***] = 0.010 mM, [Cu(NO_3_)_2_] = 0.010 mM, [ATP] = 0 - 4.0 mM.

**Table S9.** Complex formation constants of **Cu•1*α***, **Cu•1*β***, and **Cu•1*γ*** to ATP.

| Probe | Complex formation constants / M^-1^ |
| --- | --- |
| **Cu•1*α*** | (1.7 ± 0.06) × 10^3^ |
| **Cu•1*β*** | (3.3 ± 0.15) × 10^3^ |
| **Cu•1*γ*** | (2.3 ± 0.12) × 10^3^ |

**10. The competitive binding experiments of Cu•1*β*.**

**Figure S27.** Selectivity of **Cu•1*β*** for ATP in UV-Vis responses toward anions in water, pH 6.5 adjusted by PIPES/NaOH buffer, at 25 ºC. [**1*β***] = 0.010 mM, [Cu(NO_3_)_2_] = 0.010 mM, [phosphate anion] = 1.0 mM. The gray bar represents the absorbance difference upon addition of each anion (100 equiv.). The black bar represents the absorbance difference upon addition of ATP (100 equiv.) and each anion (100 equiv.).

**Figure S28.** Selectivity of **Cu•1*β*** for ATP in fluorescence responses toward anions in water, pH 6.5 adjusted by PIPES/NaOH buffer, at 25 ºC (*λ*_ex_ = 408 nm). [**1*β***] = 0.010 mM, [Cu(NO_3_)_2_] = 0.010 mM, [phosphate anion] = 1.0 mM. The gray bar represents the ratio of fluorescence intensity upon addition of each anion (100 equiv.). The black bar represents the ratio of fluorescence intensity upon addition of ATP (100 equiv.) and each anion (100 equiv.).

**11. NMR analyses of Zn•1*β*.**


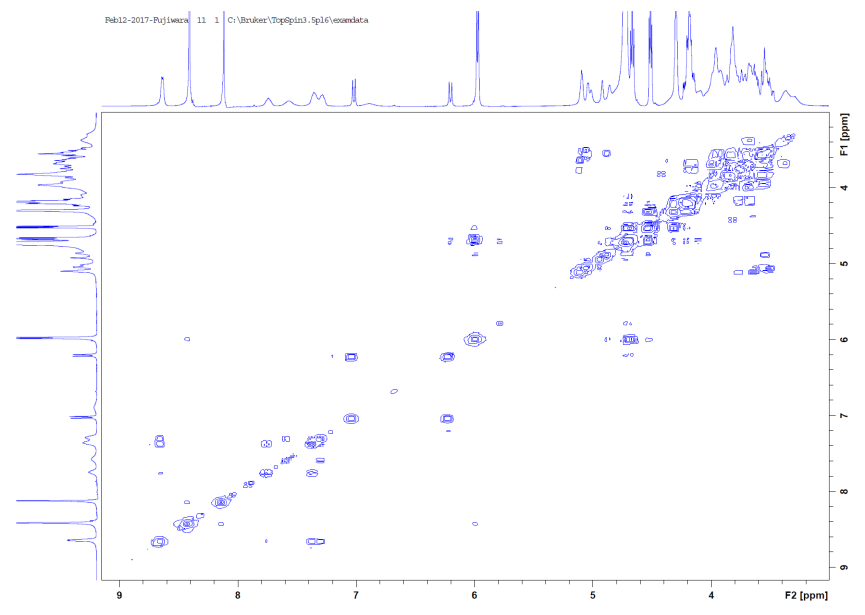


i, a

g f h

b

c

α β

H1

ε ζ η θ

H2, 4

H3, 5, 6

γ

DOH

**Figure S29.** ^1^H-^1^H COSY spectrum of the complex ATP/**Zn•1*β*** (400 MHz, solvent: D_2_O, scans: 128). [**1*β***] = 5.0 × 10^-3^ M, [Zn(NO_3_)_2_] = 5.0 × 10^-3^ M, [ATP] = 2.5 × 10^-2^ M, pD ≈ 7 adjusted by NaOD.


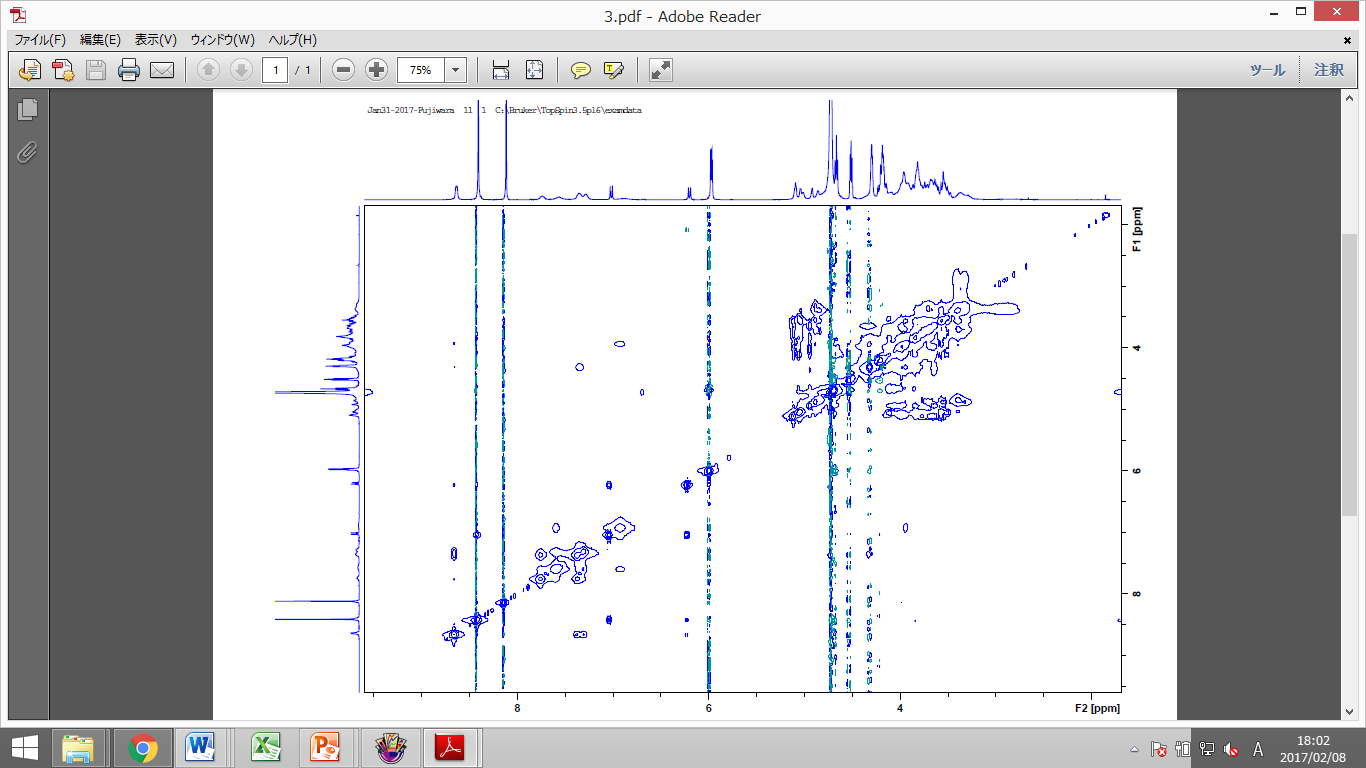


i, a

g, f, h

b

c

α β

γ

H1

ε ζ η θ

DOH

H2, 4

H3, 5, 6

**Figure S30.** NOESY spectrum of the complex ATP/**Zn•1*β*** (400 MHz, solvent: D_2_O, scans: 160, mixing time: 0.5 s). [3-dpa-HC-*β*-CyD] = 5.0 × 10^-3^ M, [Zn(NO_3_)_2_] = 5.0 × 10^-3^ M, [ATP] = 2.5 × 10^-2^ M, pD ≈ 7 adjusted by NaOD.

Zn^2+^

^-^

**Figure S31.** The NMR assignments of **Zn•1*β*** and ATP.
